# Indium-free, highly transparent, flexible Cu_2_O/Cu/Cu_2_O mesh electrodes for flexible touch screen panels

**DOI:** 10.1038/srep16838

**Published:** 2015-11-19

**Authors:** Dong-Ju Kim, Hyo-Joong Kim, Ki-Won Seo, Ki-Hyun Kim, Tae-Wong Kim, Han-Ki Kim

**Affiliations:** 1 Kyung Hee University, Department of Advanced Materials Engineering for Information and Electronics, 1 Seocheon, Yongin, Gyeonggi-do 446-701, Republic of Korea; 2 Samsung Display, OLED R&D Center, Yongin, Gyeonggi-do 446-711, Republic of Korea

## Abstract

We report on an indium-free and cost-effective Cu_2_O/Cu/Cu_2_O multilayer mesh electrode grown by room temperature roll-to-roll sputtering as a viable alternative to ITO electrodes for the cost-effective production of large-area flexible touch screen panels (TSPs). By using a low resistivity metallic Cu interlayer and a patterned mesh structure, we obtained Cu_2_O/Cu/Cu_2_O multilayer mesh electrodes with a low sheet resistance of 15.1 Ohm/square and high optical transmittance of 89% as well as good mechanical flexibility. Outer/inner bending test results showed that the Cu_2_O/Cu/Cu_2_O mesh electrode had a mechanical flexibility superior to that of conventional ITO films. Using the diamond-patterned Cu_2_O/Cu/Cu_2_O multilayer mesh electrodes, we successfully demonstrated TSPS of the flexible film-film type and rigid glass-film-film type TSPs. The TSPs with Cu_2_O/Cu/Cu_2_O mesh electrode were used to perform zoom in/out functions and multi-touch writing, indicating that these electrodes are promising cost-efficient transparent electrodes to substitute for conventional ITO electrodes in large-area flexible TSPs.

Touch screen panels (TSPs) have been considered to be key components in information devices such as mobile cellular phones, navigation systems, informative flat panel displays, and mobile pads. Among the various types TSPs, capacitive-type TSPs are the type most commonly used in mobile information devices due to their capacity for multi-touch function and multitasking as well as their easy fabrication process. However, with the emergence of flexible mobile cellular phones and curved flat panel displays, great efforts have been made to develop capacitive-type flexible TSPs^1^,^2^. To realize high-performance flexible TSPs, it is important to develop high-quality transparent and flexible electrodes because the multi-touch function or touch speed of TSPs, as well as their transparency, depend critically upon sheet resistance and optical transparency of their transparent electrodes. Although indium tin oxide (ITO) films are most commonly used as the transparent electrodes in resistive- or capacitive-type TSPs due to their high transparency and conductivity, there are critical problems making it impractical to use ITO in cost-effective flexible TSPs, including the high sheet resistance of thin ITO films, the scarcity of indium resources and thus high cost of ITO, and the poor mechanical properties of ITO films^3^,^4^,^5^. To replace conventional high-cost ITO films, several transparent electrode materials have been investigated for use as cost-effective transparent electrodes in TSPs, including carbon nanotube (CNT) network, graphene film, and conducting polymer film^6^,^7^,^8^,^9^,^10^. However, these transparent electrodes have yielded only modest performance in TSPs due to the relatively high sheet resistance of CNTs or graphene and the instability of conducting polymers. In addition, metal nanowire (NW) percolating networks and metal grid electrodes based on Ag or Cu have also been intensively investigated due to their low resistivity and superior flexibility^11^,^12^,^13^,^14^,^15^,^16^. However, the poor adhesion of Ag NW networks, their non-uniform topography, easy degradation, and their instability against static electricity is a critical problem for Ag NW network electrodes^14^. In the case of metal (Ag or Cu) grid electrodes, the resistivity is very low (2.0–4.2 × 10^−5^ Ohm-cm) but the use of highly reflective metal leads to visibility problems^17^,^18^. Although Kim *et al.* reported the low sheet resistance (6.197 Ohm/square) and high transmittance (90.65%) of Cu honeycomb mesh covered by Al-doped ZnO film, the high reflectivity of Cu metal grid is still problem to use as transparent electrodes for TSPs^19^. Recently, oxide-metal-oxide (OMO) multilayer electrodes have emerged as promising transparent electrodes for flexible organic light emitting diodes, flexible organic solar cells, flexible TSPs, flexible memory devices, and flexible oxide thin films transistors due to its low resistivity, high transmittance and good flexibility due to their low resistivity, high transparency and mechanical flexibility^20^,^21^,^22^,^23^,^24^,^25^,^26^,^27^. However, OMO multilayer films such as, ITO/Ag/ITO, IZO/Ag/IZO, and IZTO/Ag/IZTO still contain the high-cost elements of indium and silver. Although these Ag-based OMO electrodes have been extensively explored due to the very low resistivity caused by the Ag interlayer and the high transmittance caused by the antireflective effect of the dielectric/metal/dielectric structure, there have been no reports on the use of mesh-patterned OMO multilayers as transparent and flexible electrodes for flexible TSPs. In particular, the development of indium-free Cu-based OMO multilayers of mesh structure is imperative to substitute conventional high-cost ITO- or Ag-based OMO multilayers to enable cost-effective flexible capacitive-type TSPs.

In this work, we investigated the electrical, optical, and mechanical properties of mesh-patterned Cu_2_O/Cu/Cu_2_O multilayer electrodes grown by using roll-to-roll (RTR) sputtering and RTR-based wet-patterning at room temperature. By wet-patterning of Cu_2_O/Cu/Cu_2_O multilayer as a diamond-type mesh structure, we fabricated a transparent Cu_2_O/Cu/Cu_2_O mesh electrode with sheet resistance of 38 Ohm/square and optical transmittance of 90%. To our knowledge, this is the first report on the use of Cu-based OMO multilayer mesh electrode for flexible TSPs. The capacitive-type flexible TSPs with Cu_2_O/Cu/Cu_2_O multilayer grid were successfully operated, thereby demonstrating the possibility of using cost-effective Cu_2_O/Cu/Cu_2_O mesh electrodes to replace conventional high-cost ITO electrodes or Ag-based OMO electrodes.

## Results

**Figure 1:**
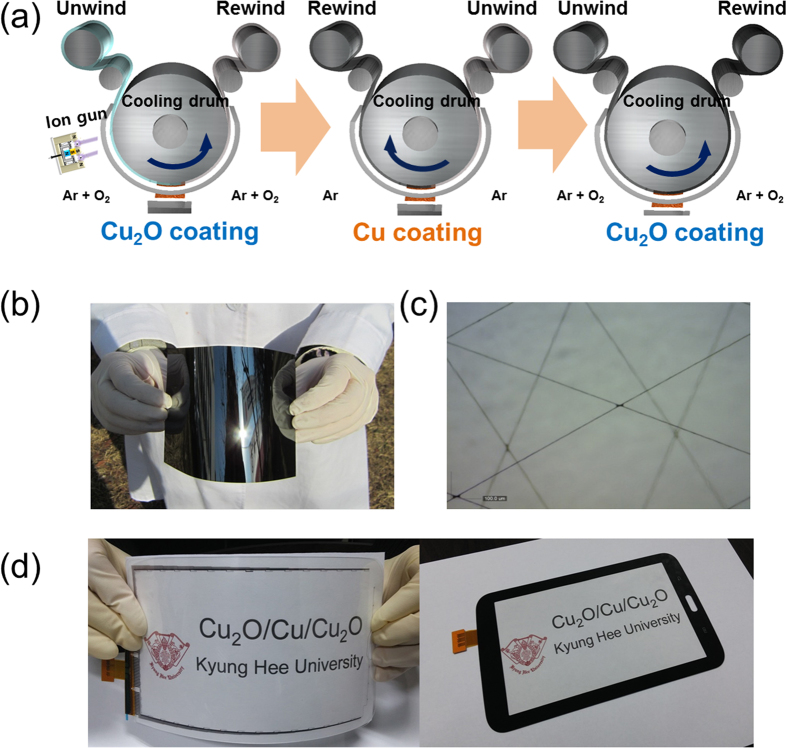
(**a**) Schematic illustration of the continuous RTR sputtering process used to fabricate Cu_2_O/Cu/Cu_2_O multilayer films on a PET substrate. (**b**) Image of brown-black Cu_2_O/Cu/Cu_2_O multilayer electrode before the mesh-patterning. (**c**) Optical microscope image of merged top and bottom Cu_2_O/Cu/Cu_2_O mesh electrodes. (**d**) Image of flexible TSPs using diamond-patterned top and bottom mesh electrodes of line width 5 μm and spacing 450 μm, before and after attachment of cover glass.

Figure 1a schematically illustrates the continuous RTR sputtering process used to deposit the bottom Cu_2_O, Cu interlayer, and top Cu_2_O film onto a flexible PET substrate without breaking vacuum. Using a pilot-scale RTR sputtering system (Figure S1), the Cu_2_O/Cu/Cu_2_O multilayer was deposited onto a PET substrate 250 mm wide by using a rectangular Cu metal target under Ar/O_2_ ambient for the Cu_2_O layers and Ar ambient for the Cu interlayer. For simplicity, we refer to the Cu_2_O/Cu/Cu_2_O multilayer films as OCO films hereafter. Figure 1b shows the resulting brown-black OCO multilayer film; it had the very low sheet resistance of 0.2 Ω/square and the resistivity of 5.9 × 10^−5^ Ω-cm prior to mesh patterning. Figure 1c shows an optical microscope image of the merged top and bottom OCO mesh electrodes having diamond-shaped patterns; these merged electrodes were used to fabricate flexible TSPs. Unlike the black color of the as-deposited OCO multilayer films in Fig. 1b, the mesh-patterned OCO electrodes were highly transparent due to their very thin mesh grid, which was approximately 5 μm wide. Figure 1d shows flexible TSP with transparent OCO mesh electrodes. If the top protective glass was removed, the TSPs could operate as flexible TSPs because both the top and bottom OCO/PET films had a good flexibility (Fig. 1d, left).

**Figure 2:**
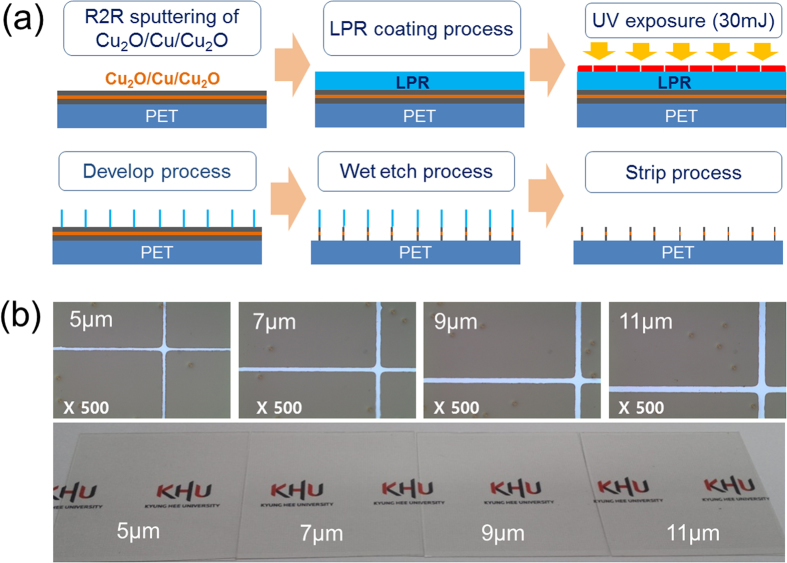
(**a**) Schematic illustration of the mesh patterning process used to form OCO multilayer films of various mesh grid line widths. (**b**) Microscope images of the OCO multilayer meshes of various line widths and pictures demonstrating transparency of OCO mesh electrodes.

Figure 2a schematically illustrates the mesh-patterning process of RTR-sputtered OCO multilayer films by using a RTR-based wet-etching system (Figure S2). By RTR coating of a liquid photo resist (LPR) layer and UV exposure of the positive-masked LPR/OCO/PET films, we successfully patterned mesh-structured OCO multilayer films of various mesh grid line widths from 5 to 11 μm (Fig. 2b). Because of dark color of the Cu_2_O layer, there was no glittering from the OCO mesh electrode, unlike Ag or Cu metal mesh grid electrodes. The bottom picture in Figure 2b clearly shows the high transparency of the mesh-patterned OCO films for all line widths below 11 μm. The continuous RTR sputtering and RTR-based wet-etching process of the OCO multilayer films indicates that the fabrication process of OCO multilayer grid electrode is well compatible to current ITO electrode fabrication process.

**Figure 3:**
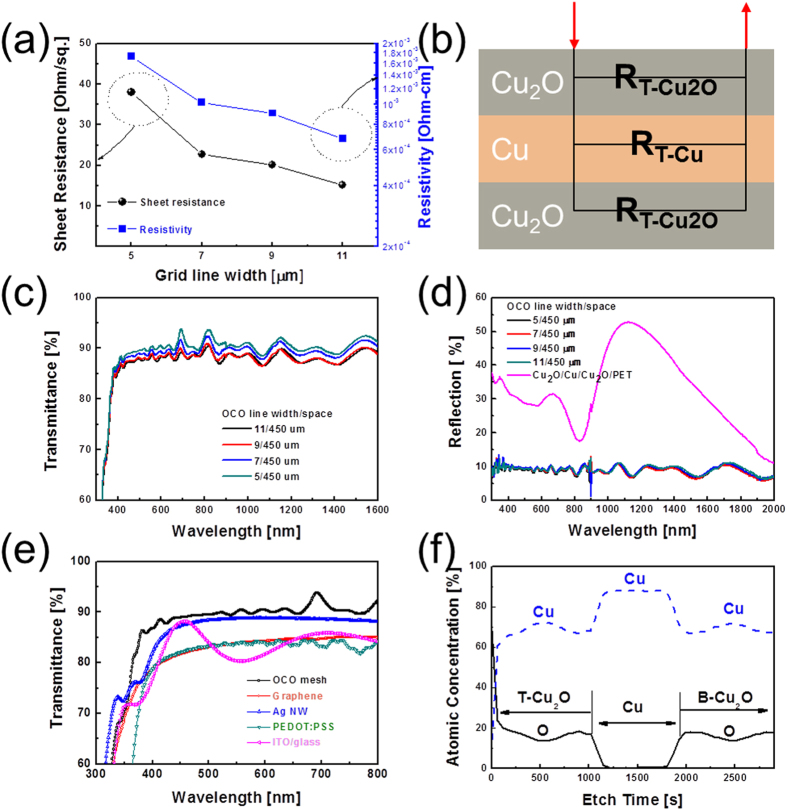
(**a**) Sheet resistance and resistivity of OCO multilayer mesh electrodes versus their grid line widths. (**b**) Schematic circuit of OCO multilayer mesh electrode. (**c**) Optical transmittance and (**d**) reflectance of OCO multilayer mesh electrodes having various line widths. (**e**) Comparison of optical transmittances of several transparent electrodes with the OCO mesh electrode. (**f**) XPS depth profile of OCO multilayer.

Figure 3a shows Hall measurement results obtained from mesh-patterned OCO multilayer electrodes of various line widths. The sheet resistance and resistivity of the OCO mesh electrodes significantly decreased as the line width was increased from 5 to 11 μm. The OCO mesh electrode of line width 11 μm had the lowest sheet resistance of 15.1 Ω/square and the resistivity of 6.8 × 10^−4^ Ω-cm, due to the presence of the metallic Cu interlayer. However, compared to the as-deposited OCO films before wet-patterning, the mesh-patterned OCO electrodes showed increased resistivity and sheet resistance because most of the Cu layer was removed by wet-etching process to increase the transparency of the OCO multilayer. Although the resistivity of the mesh-patterned OCO electrodes was higher than that of Ag or Cu metal grids due to the presence of the top and bottom semiconducting Cu_2_O layer in the OCO multilayer, the sheet resistance was acceptable for the fabrication of large area flexible TSPs above 40 inches^1^,^17^,^18^,^19^. Assuming that the total resistance of the OCO mesh electrode can be represented simply as resistances coupled in parallel of the bottom Cu_2_O, Cu, and top Cu_2_O layers (Fig. 3b). The main conduction path in this OCO multilayer electrode is likely to be the metallic Cu interlayer, similar to previously reported OMO multilayer electrodes^20^,^21^,^22^. To elucidate the electrical contribution of the Cu layer in the as-deposited OCO multilayer, the sheet resistance and resistivity of inserted thin Cu layer were extracted using the following equation (1) and (2).


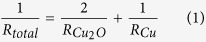



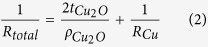


where, the R_total_, R_Cu2O_, and R_Cu_ are the sheet resistance of multilayer, Cu_2_O, and Cu, respectively. Likewise, t and ρ are the thickness and resistivity of the films. Assuming that the R_total_ of the as-deposited OCO multilayer resulted from the resistance of the single bottom-Cu_2_O (R_B-ITO_: 34.53 Ohm-cm), the Cu layer (R_Cu_) and top-Cu_2_O (R_T-ITO_) layers coupled in parallel as shown in the inset of Fig. 3b, it is possible to calculate the resistivity of the Cu interlayer. The calculated resistivity of the inserted Cu interlayer was found to be 3.0 × 10^−6^ Ohm-cm, which is sligh higher than that of bulk Cu (1.7 × 10^−6^ Ohm-cm). Therefore, it was evident that the insertion of the Cu layer significantly decreased the total sheet resistance and resistivity of the OCO multilayer electrode. Thus, the electrical properties of the OCO mesh electrode would be mainly affected by the electrical properties of the Cu interlayer. Figure 3c,d shows the optical transmittance and reflection of the mesh-patterned OCO electrodes on PET substrate versus their line width. Table 1 summarizes the optical properties of the mesh-patterned OCO electrodes. Although the optical transmittance of these OCO electrodes slightly decreased with increasing mesh grid width, all OCO multilayer mesh electrodes showed high optical transmittance in the visible wavelength region, sufficient for use in the fabrication of flexible and large-area TSPs. The mesh-patterned OCO electrodes showed peak transmittance of 90% at 550 nm and average transmittance of 89% over the visible wavelength range from 380 to 780 nm. It was noteworthy that the optical transmittance of the OCO multilayer mesh electrodes are very consistent over the entire visible range. In general, transparent OMO multilayer electrodes show significantly reduced transmittance in the near IR region due to the severe reflection from the metal interlayer^20^,^21^. However, the mesh-patterned OCO multilayer electrode showed high NIR transmittance even though it contained a Cu metal layer. Figure 3d shows the reflectance spectra obtained from a patterned OCO mesh electrode and an as-deposited OCO multilayer. As expected based on the optical transmittance results, all mesh-patterned OCO multilayer electrodes showed the low reflection of 8%, much lower than the 28.5% reflection of unpatterned OCO films (Fig. 1b). The reflection of the as-deposited unpatterned OCO films could be attributed to the reflection of the Cu interlayer. In general, the high reflection from Ag, Al, or Cu metal layer led to glittering of the metal grid electrodes. However, low reflection of the patterned OCO multilayer caused by black Cu_2_O layer indicated that there is no glittering from OCO electrodes unlike conventional metal Ag or Cu grid electrodes. Based on the sheet resistances and average transmittances of the OCO mesh electrodes, the optimum mesh grid width for obtaining a high-quality OCO mesh electrode can be determined. The figure of merit (T^10^/R_sheet_) of the OCO multilayer mesh electrode was calculated based on the average optical transmittance (T) and sheet resistance (R_sheet_)^28^. The maximum figure of merit (18.6 × 10^−3^ Ω^−1^) was obtained for the OCO mesh grid of line width 11 μm. This is comparable to the previously reported figure of merit of 24.7 × 10^−3^ Ω^−1^ for an ITO/Ag/ITO layer grown on a glass substrate^20^. Figure 3e compares the optical transmittance of the OCO mesh grid electrode to several other transparent electrodes; transferred graphene, Ag nanowire network, conducting polymer (PEDOT:PSS), and crystalline ITO/glass. At 550 nm wavelength, the mesh-patterned OCO electrode showed higher optical transmittance than all four of comparison. Figure 3f shows a XPS depth profile obtained from an OCO electrode grown on PET substrate, clearly showing its simple composition of the cost-effective elements of copper and oxygen. The XPS depth profile clearly shows that the individual top Cu_2_O layer, Cu interlayer, and bottom Cu_2_O layer were well defined on the PET, without interfacial reaction between the Cu and Cu_2_O layers. The bottom and top Cu_2_O layers were symmetrical, indicating that these layers had same thickness and composition due to the exact control over the RTR sputtering process. The binding energy of Cu 2*p*_1/2_ (951.5 eV) and 2*p*_3/2_ (931.7 eV) indicated that the RTR reactive sputtered from the Cu metal target under Ar/O_2_ ambient was the cuprous oxide (Cu_2_O) phase, as confirmed by XPS and X-ray diffraction examinations (Figure S3)^29^,^30^,^31^,^32^,^33^.

**Figure 4:**
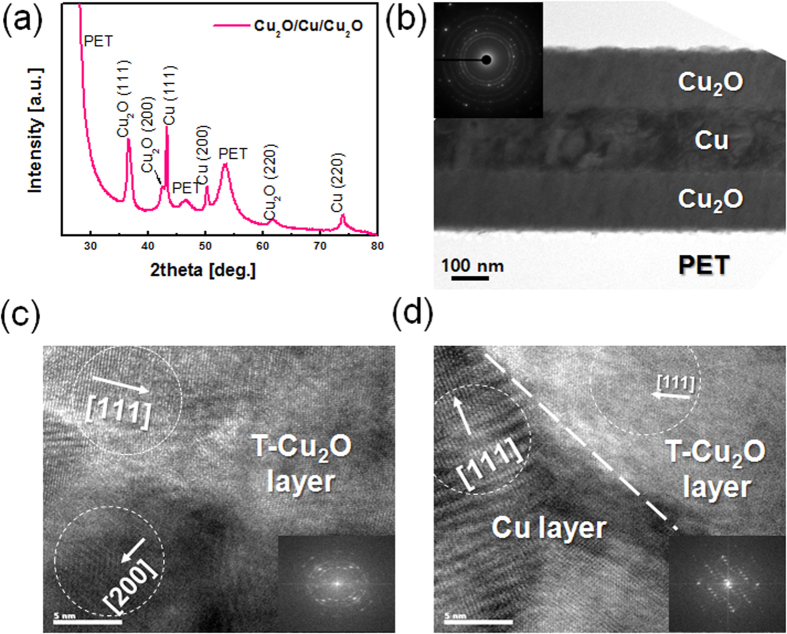
(**a**) Synchrotron X-ray scattering plot of the OCO multilayer electrode. (**b**) Cross sectional TEM image of the OCO multilayer electrode with inset of FFT pattern. (**c–d**) HRTEM images obtained from the top Cu_2_O layer and the top Cu_2_O/Cu interfacial region.

The microstructure of mesh-patterned OCO electrode was examined by synchrotron X-ray scattering (XRS) and transmission electron microscopy (TEM). Figure 4a shows the synchrotron XRS plots obtained from an OCO multilayer electrode on a PET substrate, including crystalline Cu_2_O (111), (200), and (220) peaks as well as Cu (111) and (200) peaks. The crystalline phase of the Cu_2_O films was cuprous oxide, which is the favored phase during reactive sputtering of Cu under oxygen ambient^31^,^32^,^33^. Reactive sputtered Cu could form two different oxides, such as cuprous oxide (Cu_2_O) and cupric oxide (CuO), depending on the oxygen flow ratio: Due to the low oxygen flow ratio during RTR sputtering, the reactive sputtered Cu formed a cuprous oxide on the PET substrate even though it was prepared at room temperature. Figure 4b shows cross-sectional TEM images of an OCO multilayer electrode; these images clearly demonstrate the well-defined bottom Cu_2_O (150 nm), Cu (150 nm), and top Cu_2_O (150 nm) layers, without interface layers, as expected based on the results of XPS depth profiling. These sharp interfaces indicated that there was no interfacial reaction or formation of an interfacial oxide layer between the Cu_2_O and Cu layers, which was attributed to the use of a continuous RTR sputtering process without breaking the vacuum. The symmetric structures of the bottom and top Cu_2_O layers indicated that the thickness of the Cu_2_O was precisely calibrated by controlling the rolling speed during the RTR sputtering. A fast Fourier transform (FFT) pattern in inset of Fig. 4b showed a weak circle and strong spots, which were attributed to polycrystallines Cu_2_O and Cu layers^31^,^32^,^33^,^34^,^35^. Figure 4c is a HRTEM image obtained from the top Cu_2_O (T-Cu_2_O) layer. As expected based on the XRS plot, the T-Cu_2_O layer was polycrystalliens with (111) and (200) preferred orientations. Even though the T-Cu_2_O layer was sputtered at room temperature, it showed a well-developed polycrystalline structure. The bright region in Fig. 4c clearly showed the existence of a (111) preferred crystalline Cu_2_O phase in the T-Cu_2_O layer. The FFT pattern in the inset of Fig. 4c also showed strong spots and circles, indicating a polycrystalline Cu_2_O cuprous phase with (111) and (200) preferred orientations. Figure 4d is a HRTEM image obtained from the interface between the T-Cu_2_O and the Cu interlayer; this image shows the well-defined interface between the Cu metal layer and the Cu_2_O semiconductor layer (indicated by a dashed line in the figure), attributed to the room-temperature RTR sputtering process carried out without breaking vacuum. As discussed by Alford *et al.* the metallic layer in an OMO multilayer acts as an electron source for oxide layer; therefore, the Cu interlayer, which was in good contacted with the Cu_2_O layer could provide electrons to and increase the carrier concentration of the Cu_2_O layer^27^.

**Figure 5:**
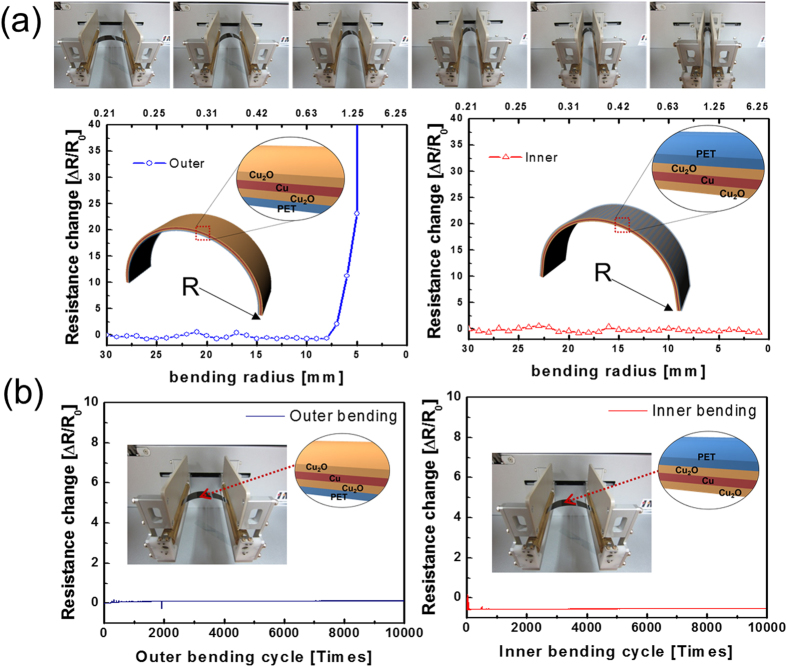
(**a**) Outer and inner bending test results of the OCO electrode on flexible PET substrate for various bending radii. Inset illustrated OCO/PET films under outer and inner bending. Upper panels show outer and inner bending steps of OCO/PET films. (**b**) Dynamic outer and inner fatigue test comprising 10,000 cycles at constant bending radius of 10 mm.

Figure 5a shows the results of outer/inner bending tests of OCO multilayer films for various outer/inner bending radii. The change in the resistance of the OCO multilayer electrode that results from bending can be expressed as (R-R_0_)/R_0_, where R_0_ is the initial measured resistance and R is the resistance measured under substrate bending^22^,^26^. The upper panel of the Fig. 5 shows pictures of the outer/inner bending test steps with decreasing bending radius. The outer bending test results in Fig. 5a showed that the OCO multilayer had constant resistance until the bending radius reached 7 mm. Based on the following equation, we can calculate the peak strain for the curved OCO multilayer film with decreasing bending radius^22^,^26^.


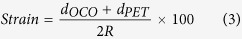


Here, *d*_OCO_ and *d*_PET_ are the thicknesses of the OCO multilayer and the PET substrate, respectively. Bending a 450-nm-thick OCO film on a 125-μm-thick PET substrate to the bending radius of 7 mm resulted in a peak strain of 0.95%. Further decreasing the outer bending radius rapidly increased in the resistance change due to crack formation and propagation in the top Cu_2_O layer. In the inner bending tests, the measured resistance of the OCO multilayer film was constant until the sample was bent to an inner bending radius of 2 mm (the bending limit); at this radii, the OCO multilayer experienced the peak strain of 3.12%. Even though the OCO films delaminated from the PET substrate or many cracks formed in the OCO films under this condition, the change of resistance was very small. Under compressive stress, the flexible OCO film remained functional despite the local delamination of the layer or crack formation, due to overlapping of cracked or delaminated layers. However, when outer bending was applied, the OCO films were under tensile stress, as shown in the inset of Fig. 5a. Due to this tensile stress, cracks formed and propagated. Therefore, cracks isolated the OCO multilayer and increased the resistance change when it was severely bent below the bending radius of 7 mm. Figure 5b shows the dynamic outer and inner bending test results of the optimized OCO multilayer mesh electrode sample with increasing bending cycles at a fixed inner bending radius of 10 mm. The bending radius of 10 mm is an acceptable degree of bending for application in flexible TSPs. Both dynamic outer bending fatigue tests showed no change in resistance (ΔR) after 10,000 bending cycles, demonstrating the superior flexibility of the OCO multilayer. This superior flexibility can be attributed to the high resistance to strain failure of the metallic Cu interlayer between the Cu_2_O layers^12^.

**Figure 6:**
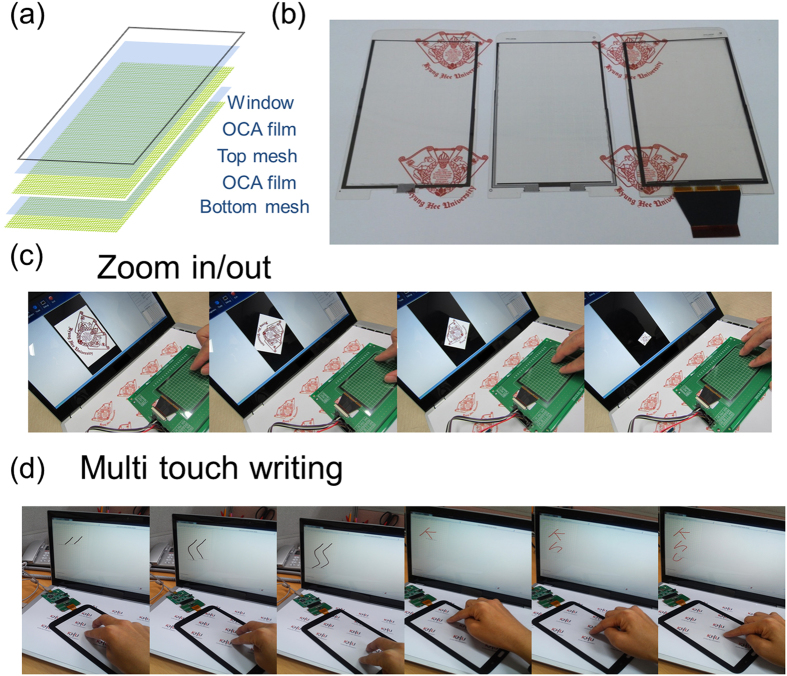
(**a**) Schematic structure of GFF-type TSPs with top and bottom multilayer grid electrodes based on OCA film. (**b**) Picture of bottom OCO/PET, top OCO/PET, and merged OCO mesh electrode with grid line width 5 μm, used for fabrication of GFF-type TSPs. (**c**) Zoom in and out function of GFF-type flexible TSPs fabricated on a diamond-patterned OCO multilayer mesh electrode. (**d**) The multi-touch writing and single-touch writing functions of the TSP with a glass cover layer.

Figure 6a shows the schematic structure of glass-film-film (GFF)-type TSPs with diamond-shaped top and bottom OCO mesh electrodes. By applying optical clear adhesive (OCA) films, the top OCO mesh electrode films could be attached to the bottom OCO mesh electrode. Figure 6b shows a picture of the top OCO/PET films, the bottom OCO/PET film, and the merged OCO/OCA/OCO films with diamond-shaped mesh of line width 5 μm used to fabricate TSPs. By connecting the GFF-type TSPs to software, we were able to operate the TSP based on the diamond-patterned OCO multilayer mesh electrode (Figure S4). Figure 6c shows the zoom in and zoom out functions of the flexible TSP fabricated based on the diamond-patterned OCO multilayer mesh electrode. Generally, the GFF-type TSP was operated by exact sensing of X-Y coordinates and characteristics of linearity. The TSPs with diamond-shaped OCO mesh electrodes were also operated without protective cover glass, indicating the possibility of flexible TSPs based on the OCO mesh electrodes. Figure 6d shows the multi-touch writing function of the TSP with a glass cover layer. The TSP with the diamond-patterned OCO multilayer mesh electrode was successfully used to perform zoom in, zoom out, and multi-touch writing functions. This demonstrated that the diamond-patterned OCO multilayer mesh electrode, which has low sheet resistance and high optical transmittance as well as good mechanical flexibility, is a promising transparent, flexible, and cost-effective electrode for substituting conventional ITO electrodes in large area flexible TSPs.

## Conclusion

In summary, we developed a method to fabricate indium-free and cost-effective OCO multilayer mesh electrodes by means of RTR sputtering at room temperature; these OCO electrode are a viable alternative to ITO electrodes or OMO electrodes for the low cost production of flexible TSPs. By using the low resistivity of the metallic Cu layer and patterned mesh structure, we obtained highly transparent OCO multilayer mesh electrodes with low sheet resistance. It was found that the tunable electrical and optical properties of the OCO multilayer mesh electrodes were affected by the mesh line width. Outer/inner bending test results showed that the OCO mesh electrode had mechanical flexibility superior to that of conventional ITO films. By using a diamond-patterned OCO multilayer mesh electrode, we successfully demonstrated the operation of flexible TSPs, including zoom in/out functions and multi-touch writing; this indicated that the OCO mesh electrodes are promising as substitutes for conventional ITO electrodes in large-area flexible TSPs.

## Methods

### Deposition of Cu_2_O/Cu/Cu_2_O (OCO) multilayer films

OCO multilayer films were continuously sputtered on a PET substrate of width 250 mm (Kimoto Ltd., Japan) at room temperature by using a specially designed pilot-scale RTR sputtering system (Figure S1). Prior to the sputtering of the bottom Cu_2_O layer, the surface of the PET substrate was pretreated by means of irradiation by an Ar ion beam operated at a DC pulsed power of 1.2 kW; this removed organic contamination and improved the adhesion between the bottom Cu_2_O layer and the PET substrate. After this ion treatment, the 150 nm thick bottom Cu_2_O layer was reactively sputtered onto the PET substrate by using a Cu target (460 mm × 130 mm); the operating conditions used were the DC power of 2.2 kW, working pressure of 3 mTorr, Ar/O_2_ flow rate of 400/120 sccm, and rolling speed of 0.4 m/min. After the sputtering of the bottom Cu_2_O layer, a Cu layer 150 nm thick was directly sputtered onto the bottom Cu_2_O at a constant DC power of 2.2 kW, working pressure of 3 mTorr, Ar flow rate of 450 sccm, and rolling speed of 0.4 m/min. Finally, the top Cu_2_O layer was sputtered onto the Cu layer by using conditions identical to those used for the bottom Cu_2_O layer and without breaking vacuum.

### Characterization of Cu_2_O/Cu/Cu_2_O multilayer mesh electrodes

The sheet resistance and resistivity of the OCO multilayer mesh electrodes were measured by means of Hall measurements (HL5500PC, Strength 0.32 T, Accent Optical Technology) at a room temperature. The optical transmittance of the OCO multilayer mesh electrode was measured by means of UV/visible spectrometry (Lambda 35) carried out over the spectra range from 220 to 1600 nm. The structural properties of the OCO multilayer electrode were analyzed by means of synchrotron X–ray scattering conducted at the GI-WAXS beam line of the Pohang Light Source. The microstructures and interfacial structures of the optimized OCO multilayer mesh electrodes were examined by means of high-resolution electron microscopy (HRTEM). FFT images were obtained from a cross-sectional HREM specimen prepared by means of focus ion beam (FIB) milling. In addition, the interfacial properties of the optimized OCO multilayer mesh electrodes were analyzed by using X-ray photoelectron spectroscopy (XPS) depth profiling. The mechanical properties of OCO multilayers were evaluated by using a specially designed inner/outer bending system. The outer bending test induced tensile stresses on the film, whereas the inner bending test induced compressive stress. In addition, dynamic fatigue bending tests were carried out by using a lab-designed cyclic bending test machine, operated at the frequency of 0.5 Hz for 10,000 cycles. The resistances of the OCO multilayers were measured throughout the cyclic bending.

### Roll-to-Roll patterning of OCO films and fabrication of flexible TSPs

For mesh patterning of OCO films, a liquid photo resist (LPR) layer was coated onto the RTR sputtered OCO multilayer films by using a commercial slot die coating system (DKT Ltd., Korea). Then the LPR-coated OCO films were passed over a heating chamber by means of unwinding and rewinding roller motions (Figure S5). The RTR-sputtered OCO multilayer films were patterned by using wet-patterning system (Figure S2). The LPR-coated OCO multilayer films were exposed to UV light by using a positive grid mask. The UV-exposed OCO multilayer films were patterned by a spray-type developer using a developing solution (EN-DT238E: tetramethylammonium hydroxide 3%, surfactant 2%, deionized water 95%). The patterned OCO multilayer films were subsequently etched by a spray-type wet etching system using an etching solution (0.5% FeCl_3_ in deionized water). The wet-etched OCO films were striped by a spray-type stripper system using a stripping solution (EN-S800Mo: glycol ethers 10%, sodium gluconate 10%, EDTA 10%, surfactant 5%, deionized water 65%). Finally, the stripped OCO multilayer films were cleaned by a spray-type rinse system using deionized water. The resulting diamond-patterned OCO multilayer mesh electrodes were used to fabricate film-film-type flexible TSPs and GFF-type rigid TSPs. The top OCO/PET and bottom OCO/PET films was attached to each other using an OCA film. The resulting OCO/PET/OCA/OCO/PET films were connected to a flexible printed circuit board by means of bonding both the metal pattern and the FPCB to an anisotropic conductive film (Figure S4). By attaching cover glass to the top OCO/PET films, we were able to fabricate the GFF-type rigid TSPs. Finally, the FPCB was connected to an IC controller.

## Additional Information

**How to cite this article**: Kim, D.-J. *et al.* Indium-free, highly transparent, flexible Cu_2_O/Cu/Cu_2_O mesh electrodes for flexible touch screen panels. *Sci. Rep.* **5**, 16838; doi: 10.1038/srep16838 (2015).

## Supplementary Material

Supporting Information

## Figures and Tables

**Table 1:** Optical properties of mesh-patterned OCO multilayer electrodes with increasing grid line width.

Mesh grid line/space [μm]	Transmittance at 550 nm [%]	Average transmittance in 380 ~ 780 nm [%]	Reflection at 550 nm [%]
5/450	90.0	89.9	8.2
7/450	89.4	89.0	8.5
9/450	88.6	88.2	8.8
11/450	88.1	87.9	8.8
