# Supplementary material for: Indium-free, highly transparent, flexible Cu2O/Cu/Cu2O mesh electrodes for flexible touch screen panels
Source: Sci Rep. 2015 Nov 19;5:16838. doi: 10.1038/srep16838 (PMC4652235; doi:10.1038/srep16838)
Supplement: Supporting Information [file srep16838-s1.pdf]

Additional information

Indium-free, highly transparent, flexible Cu_2O/Cu/Cu_2O mesh electrodes for flexible touch screen panels

*By Don-Ju Kim[1], Hyo-Joong Kim[1], Ki-Won Seo[1], Ki-Hyun Kim[2], Tae-Wong Kim[2] and Han-Ki Kim[1,]**

((Optional Dedication))

[1]Kyung Hee University, Department of Advanced Materials Engineering for Information and Electronics, 1 Seocheon, Yongin, Gyeonggi-do 446-701, Republic of Korea

[2]Samsung Display, OLED R&D Center, Yongin, Gyeonggi-do 446-711, Republic of Korea

[*]imdlhkkim@khu.ac.kr

Roll-to-roll sputtering of Cu_2O/Cu/Cu_2O multilayer films: **Figure S1** shows the RTR sputtering system used to deposit Cu_2O and Cu layers on the flexible PET substrate roll. The RTR sputtering system was equipped with a rewind roller, an unwind roller, a cooling drum, and a cold cathode-type ion gun system. By using an unwinding and rewinding system, the flexible PET substrate was continuously passed over the Cu target. In addition, the tension of the flexible PET substrate was controlled by a load cell in the rolling system. The PET substrate of width 250 mm and thickness 125 μm was passed over the cooling drum, which was in mechanical contact with the substrates. The rolling speed of the PET substrate could be exactly controlled by the motor speeds of unwind and rewind roller. The Cu (99.99%, Advance Materials Ltd., Korea) target and ion gun were placed at the distance of 100 mm from the PET substrate. Prior to the RTR sputtering, the surface of the PET substrate was pre-treated by irradiation of Ar^+ ions at a constant pulsed DC power of 1.2 kW to improve the surface morphology of the PET substrate and its adhesion with the bottom Cu_2O film. After this ion-beam pretreatment of the PET substrate, a 150 nm thick bottom Cu_2O layer was sputtered at the constant Ar/O_2 flow ratio of 400/120 sccm, DC power of 2.2 kW, and working pressure of

3 mTorr by using a rectangular Cu target of geometry of 460 mm×130 mm placed below the rotating cooling drum at a distance of 100 mm.

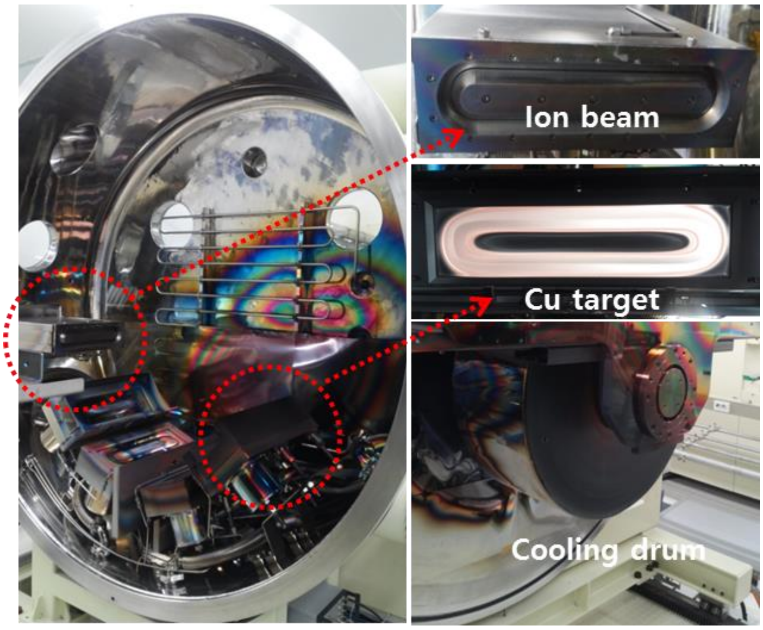

Figure S1. Pictures of a roll-to-roll (RTR) sputtering system equipped with a linear ion beam gun and linear Cu target.

The thickness of the Cu_2O layer could be controlled by the rolling speed of the PET substrate as it passed over the Cu target. After sputtering of the bottom Cu_2O layer, a 150 nm thick Cu layer was then RTR-sputtered onto the bottom Cu_2O layer by using the constant Ar flow of 450 sccm, DC power of 2.2 kW, and working pressure of 3 mTorr, using the same Cu target. The thickness of the Cu interlayer was also controlled by the rolling speed of the PET substrate. Finally, a top Cu_2O layer was sputtered onto the Cu interlayer under deposition conditions identical to those used to sputter the bottom Cu_2O layer. As illustrated in **Figure S1**, the multilayer was sputtered onto the PET substrate in a single chamber by using a winding and rewinding system.

Diamond-shaped mesh patterning of OCO multilayer films: **Figure S2** shows the wet etching

system that was used for mesh patterning of OCO multilayer films. Exposed OCO multilayer films was developed by using a spray-type developing system operated at room temperature using the line speed of 1m/min, and the pressure of 1 kgf/cm^2 , and using a developing solution (EN-DT238R ; Tetramethylammomium hydroxide - 3%, surfactant - 2%, deionize water - 95%). The developed OCO multilayer films were subsequently etched by using a spray-type etching system operated a room temperature, using the line speed of 2m/min, and the pressure of 2 kgf/ cm^2, and using etching solution.

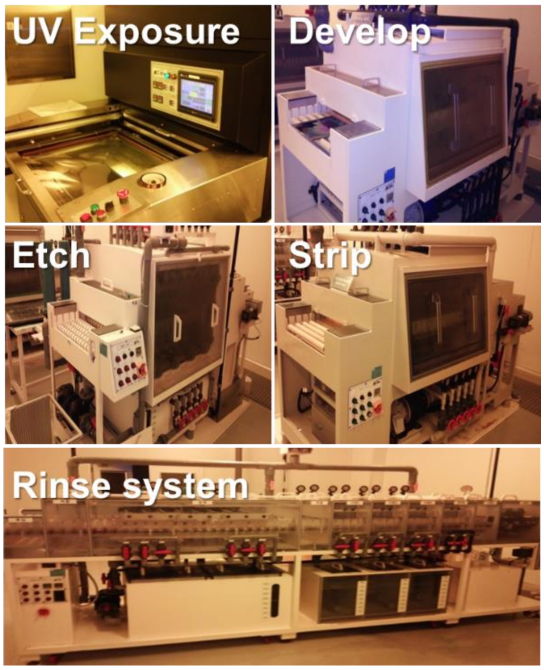

Figure S2. Picture of the UV exposure, developer, etcher, striper, and rinsing systems for diamond-shaped patterning of OCO multilayer films..

Etched OCO multilayer films were stripped by a spray-type strip system using the line speed of 4m/min, and pressure of 4 kgf/ cm^2 , and using a striping solution (EN-S800 Mo ; glycol ethers 10%, EDTA -10%, surfactant-5%, sodium gluconate -10%, deionized water -65%). Finally, stripped OCO multilayer films were cleaned by a spray-type rinsing using the line speed of 2 m/min, and the pressure of 2 kgf / cm^2, and using a deionized water.

RTR sputtered Cu_2O films: To confirm the Cu_2O phase prepared by RTR sputtering, XRD examination for the Cu_2O/PET sample was carried out. The XRD plot in **Figure S3**a showed crystalline Cu_2O peaks at 2θ = 36.6° (111), 42.5° (200), and a board peak of PET substrate. As discussed by Pierson *et al.*, the direct optical bandgap energy of the Cu_2O ranges from 2.10 to 2.6 eV, whereas the bandgap of the CuO phase ranges from 1.3 to 2.1 eV.[1,2] As shown in **Figure S3**b, the direct optical bandgap of the RTR sputtered Cu_2O film was 2.4 eV, indicating the cuprous oxide (Cu_2O) phase.

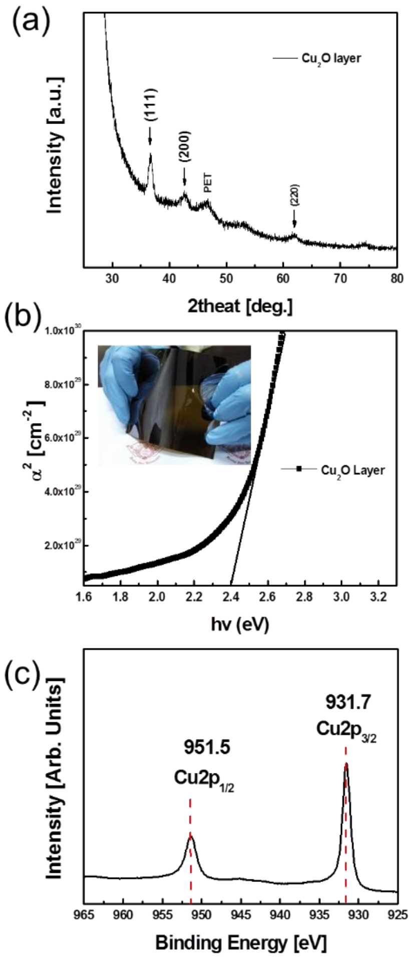

Figure 3S. (a) XRD plot of RTR-sputtered Cu_2O film and (b) direct optical bandgap energy of the Cu_2O layer. (c) Core level spectra of the Cu 2*p* peaks obtained from Cu_2O layer.

Figure S3c shows the XPS core level spectra of the Cu2p peaks obtained from RTR sputtered

Cu_2O film. The binding energies of Cu2$p_{1/2}$ (951.5 eV) and 2$p_{3/2}$ (931.7 eV) obtained from the top Cu_2O film were well matched with the cuprous Cu_2O phases.[3,4] Based on the results of the XRD and XPS examinations, we concluded that the RTR sputtered films were of the cuprous oxide (Cu_2O) phase.

Fabrication and operation of TSPs with mesh patterned OCO electrode: To investigate the feasibility of using the OCO multilayers as transparent electrodes for FF- or GFF-type TSPs, we fabricated simple GFF-type TSPs by using the diamond-patterned OCO multilayer electrodes. **Figure S4** shows the top OCO multilayer, bottom OCO multilayer and merged GFF-type TSPs. Both top OCO/PET and bottom OCO/PET films were bonded to a flexible printed circuit board (FBCB) using an anisotropic conductive film, and then cover glass was attached to the top OCO multilayer by using OCA film to protect the patterned OCO multilayer. Finally, the FPCB was connected to an IC controller to operate TSPs..

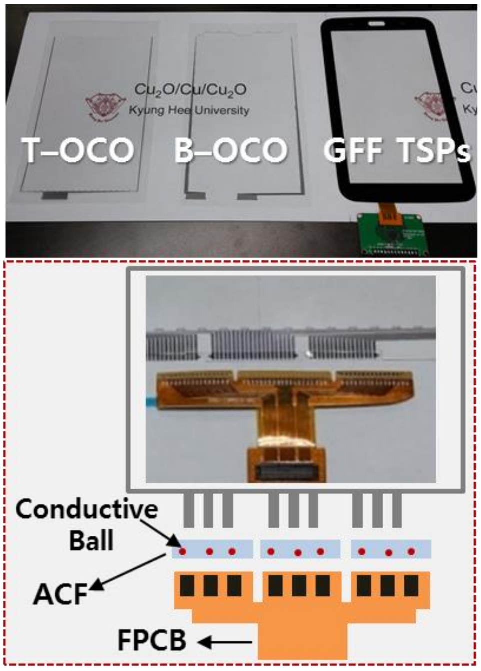

Figure S4. Picture o FF- and GFF-type TSPS fabricated on the diamond-shped patterned OCO multilayer electrode. Connection of TSPs to FPCB and IC controller.

LPR coating process using slot die coating system: To fabricate mesh-patterned OCO electrodes, positive LPR (AZ SOP-53, AZ Electronic Materials Ltd. in USA) was coated onto the OCO multilayer films by using a commercial slot-die coating system (DKT Ltd., Korea) as shown in **Figure S5**. This system was equipped with an unwind roller, rewind roller, slot die coater, tension controller, and heating chamber zone (8m). After the LPR coating, the films were passed over the 8m-heating zone of heating temperature 95 ℃ at the speed of 0.8 m/min by motion of the unwind and rewind roller.

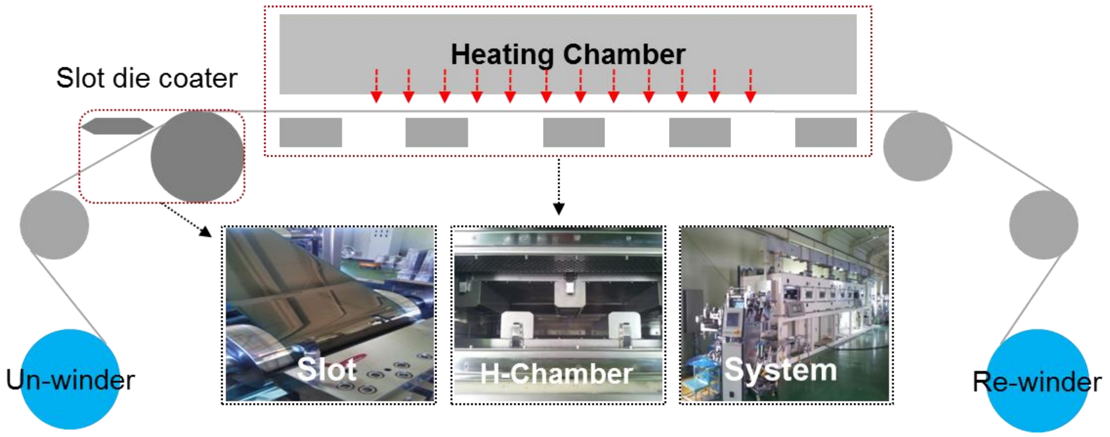

Figure S5. Schematic and picture of RTR slot die coating system with substrate heating chamber.

[1] B. Balamurugan, B.R. Mehta, *Thin Solid Films* **2001**,396,90.

[2] J.F. Pierson, A. Thobor-Keck, A.Billard, *Appl. Sur. Sci.* **2003**,210,359.

[3] D. Tahir, S. Tougaard, *J. Phys. : Cond. Matt.* **2012**,24,175002

[4] J.P. Espinos, J.Morales, A. Barranco, A. Caballero, J.P. Holgado, and A.R. Gonzalez-Elipe, *J. Phys. Chemistry B* **2002**,106,6921.
